# Serial Foodborne Norovirus Outbreaks Associated with Multiple Genotypes

**DOI:** 10.1371/journal.pone.0063327

**Published:** 2013-05-08

**Authors:** Jianwei Huang, Xuerong Xu, Qinyun Weng, Huarong Hong, Zhinan Guo, Shuizhen He, Jianjun Niu

**Affiliations:** Xiamen Center for Disease Control and Prevention, Xiamen, Fujian Province, China; Wadsworth Center, United States of America

## Abstract

Noroviruses (NoV) have been recognized as an important pathogen associated with acute gastroenteritis worldwide during the past three decades. In the spring of 2012, a series of foodborne outbreaks in tourist groups were reported to Xiamen Center for Disease Control and Prevention, Xiamen, Fujian province, China. Among a total of 268 tourists in 7 groups, the prevalence rate of acute gastroenteritis was 16.0% (43/268). Twenty-three feces or anal swabs were collected for laboratory tests of causative agents, no bacterial pathogen was identified, while 22 of them were positive for NoV RNA. In addition, thirteen NoV fragments were recovered from positive specimens and sequenced, belonging to five genotypes such as GI.3, GI.4, GII.4, GII.6, and GII.14, respectively. However, NoV fragments obtained from locally infected patients showed distinct genotypes. Therefore, epidemiological investigation and laboratory analyses demonstrated that the serial foodborne NoV outbreaks in tourists were co-infection of multiple genotypes induced acute gastroenteritis linked to a restaurant.

## Introduction

Noroviruses (NoV) are leading causes of globally virus-associated acute diarrhea. Since the first identification of NoV in 1968, the majority of non-bacterial gastroenteritis cases are contributed by NoV infection, which is also well-known as stomach flu in that NoV share certain properties with influenza viruses of their rapid transmissibility, high infectivity and frequent association with large outbreaks [1], [2]. NoV belong to the genus Norovirus in the *Caliciviridae* family, which are non-enveloped RNA viruses with a single stranded positive genome ranging from 7.3 kb to 8.5 kb [3]. According to nucleotide divergence across the genome, NoV are currently divided into 5 genogroups (GI, GII, GIII, GIV and GV), three of which (GI, GII and GIV) have been documented in association with human disease. Meanwhile, several genotypes are subsequently characterized based on further diversity within each genogroup [4]. From the NoV genotyping scheme developed by Kageyama et al, which is based on variability in the N/S domain of the capsid gene, there are at least 14 genotypes in GI and 17 genotypes in GII, which has been the predominant genogroup circulating worldwide for decades [5]. Conventionally, NoV strains harboring greater than 80% sequence homology in the capsid gene, or nucleotide similarity in the polymerase region with greater than 85% in GI or 90% in GII, are classified into the same genotype [6].

With an incubation period ranged from 12–48 hours, NoV infection occurs in all age groups [7]. The transmission for NoV is mainly via the oral fecal route or direct person-to-person contact, however, it was indicated by recent epidemiological events that contaminated water and tainted foodstuff played important roles in epidemic of viral gastroenteritis, nearly 50% of NoV infections were associated with foodborne outbreaks [8]. Previous data from Center for Disease Control and Prevention, USA, revealed that out of 226 fecal specimens from non-bacterial gastroenteritis patients, 81% contained NoV RNA, including 79% GII strains and 19% GI strains [9]. A recent investigation in China also showed that nearly 26% of some 4000 fecal samples from pediatric outpatients were NoV-positive, notably, more than 98% belonged to GII [10]. Local surveillance data in 2008 from Xianmen, China, also indicated 21.05% of fecal samples collected from diarrheal patients were positive for NoV antigens, 33.13% of them were positive for NoV RNA, in which GII strains were dominant (74.8%) [11].

Between February and April, 2012, a series of acute gastroenteritis in several tourist groups were reported to Xiamen Center for Disease Control and Prevention, Xiamen, China. NoV infections were suspected from clinical features, hence, fecal specimens from symptomatic patients were collected for laboratory tests, which subsequently confirmed primary hypothesis of etiology. Since epidemiological investigation indicated the serial outbreaks in tourists were food-related, additional sequence analyses indicated at least five genotypes were identified from positive fecal specimens, the evidence chain demonstrated that the serial foodborne NoV outbreaks in tourists were co-infection of multiple genotypes that was linked to a restaurant ca 180 km away from Xiamen.

## Materials and Methods

### Background of Outbreaks

On February 9–11th, 2012, three clusters of acute gastroenteritis outbreaks in tourist groups were reported in three hospitals in Xiamen, China (Event A–C). On April 12–17th, another 4 similar outbreaks in tourist groups were reported to Xiamen Center for Disease Control and Prevention (Event D–G). It was revealed by preliminary epidemiological investigation that the seven tourist groups had lunches at the same restaurant on a coastal resort one day before their arrival at Xiamen, suggesting serial foodborne outbreaks. Hence, a field investigation on the suspicious restaurant was conducted on April 18^th^. Coincidently, another three visitors from the neighbouring Zhejiang province presented acute gastroenteritis and were hospitalized in Xiamen (Event H) on the same day.

### Sample Collection and Processing

In each event, epidemiological investigation was immediately conducted on receiving reports from hospitals. Fecal specimens from hospitalized patients were collected for laboratory tests, however, no specimens were collected from non-hospitalized patients with mild symptoms. During the field investigation for the suspected restaurant on April 18th, fourteen anal swabs were collected from its employees, along with other samples including raw oyster, cooked oyster, shrimps, crabs and raw water, etc.

Although dozens of fecal specimens or anal swabs were collected from hospitalized patients or healthy individuals for laboratory tests, the purpose of this study was to identify any causative agents associated with the serial outbreaks, and to facilitate timely taking of relevant intervention and control measures. All participants were voluntarily cooperative in providing specimens and questionnaire information during case investigation. Therefore, the present study conducted within Fujian province, China, did not involve human research, IRB review was not required.

Laboratory analyses included isolation of bacterial pathogens such as *Salmonella spp, Vibrio parahaemolyticus, Shigella spp, Staphylococcus aureus, Proteus spp* and diarrheagenic *Escherichia coli*, etc., besides detection of NoV RNA by real-time RT-PCR.

### Nucleic Acid Extraction

Suspensions for food samples were prepare according to standard protocols [12], [13]. Briefly, 5 g of digestion tracts or adjacent soft tissues were homogenized for 3 min in 35 mL of glycine buffer (pH 9.5), incubated at 37°C for 30 min or shaken for 30 min at room temperature, then centrifuged for 30 min at 10000 g, 4°C. The supernatants were thoroughly mixed with an equal volume of PEG8000 solution at a final concentration of 8%, incubated on ice for 1 hour, centrifuged for 5 min at 10000 g, 4°C, the resulting pellets were re-suspended in 2 mL of phosphate-buffered saline (PBS) for nucleic acid extraction.

Prior to RNA extraction, water samples were pretreated as previously described [14]. MgCl_2_ was added in one liter of water sample to a final concentration of 50 mmol/L, after adjusting pH value to pH 3.0 with 1 mol/L HCl, the sample was filtered with one 0.45 µm mixed nitrocellulose membrane. Subsequently, the membrane was eluted in 10 mL elution buffer (3% beef extract, 50 mmol/L glycine, pH 9.5), the virus-containing buffer was concentrated with PEG8000 as the abovementioned protocol for food samples.

Similarly, suspensions for anal swabs were prepared by adding 2 mL of PBS with brief vortexing, then centrifuged for 5 min at 8000 g, the supernatants were subjected to RNA extraction.

In the RNA extraction step, total RNA was extracted from 200 µL of pretreated supernatants by using a Roche High Pure Viral RNA Kit (Roche, Mannheim, Germany).

### Detection of NoV RNA

NoV RNA was detected with a one-step real time PCR kit for GI and GII (Liferiver Biotechnology Company, Shanghai, China), on the Applied Biosystems 7500 real-time PCR system (Applied Biosystems, Foster City, CA, USA) following the manufacturer’s instructions. The amplification condition was: 50°C 20 min; 95°C 3 min; 95°C 5 s, 60°C 40 s, 40 cycles.

### Sequencing of NoV Fragments

Primers specific for partial VP1 gene of GI and RdRP/VP1 of GII were designed according to NoV genome data available on GenBank (Table 1). Positive samples in real-time PCR assays were amplified by a PrimeScript® II High Fidelity RT-PCR Kit (TaKaRa, Dalian, China). As instructed by the manufacturer’s protocol, 0.5 µL of both sense and anti-sense primers, 5 µL of RNA templates were added in a 40 µL volume, relevant DNA fragments of GI and GII were amplified. The amplification condition was set as follows: 50°C 30 min of reverse transcription, 95°C 2 min for denaturation; 95°C 30 s, 55°C 60 s, 72°C 60 s for 35 cycles; 72°C extension for another 10 min. All amplicons were visulized on 1.5% agarose gels, then gel-purified and sequenced at a sequencing service center of Invitrogen (Shanghai, China).

**Table 1:** Primers for amplification and sequencing of partial RdRP/VP1 gene.

Genogroup	Primers	Amplicon location
NoV GI	GI-F: 5′-CTGCCCGAWTWYGTAAATGA-3′	5339∼5668 (330 bp)
	GI-R: 5′-CCAACCCARCCATTRTACA-3′	
NoV GII	GII-F: 5′-ATGTTCAGGTGGATGAGATT-3′	5012∼5387 (376 bp)
	GII-R: 5′-ACCWGCATAACCATTGTACAT-3′	

### Phylogenetic Analyses of NoV Sequences

Outbreak-associated sequences were on-line analyzed by the BLAST tool of NCBI (http://www.ncbi.nlm.nih.gov/BLAST), ten NoV fragments containing the amplified regions with high homology and distinct geographic origins were downloaded as reference sequences for alignment analyses (accession numbers: HE716747, JQ743330, JX898883, JN603244, JX488750, JQ743331, JN183165, GQ845370, HM635109 and HM635092). Briefly, these sequences were aligned by ClustalW2.0 [15], then a phylogenetic tree was constructed using the neighbor-joining method of the Molecular Evolutionary Genetics Analysis (MEGA) 5.0 package [16]. Thus, genetic relationship between NoV strains from the serial outbreaks with global circulating strains was determined.

## Results

### Clinical and Epidemiological Findings

Retrospective investigation showed similar clinical symptoms including nausea, vomiting, abdominal pain, diarrhea, dizziness and headache, etc in most patients, who had normal white cell counts, lowered lymphocyte ratios and elevated neutrophilic granulocyte ratios. These patients were eventually diagnosed as NoV-caused acute gastroenteritis in that NoV RNA was detected in most fecal specimens (Table 2). Among the 7 tourist groups, six groups were from Taiwan, one group was from Malaysia. The prevalence rate of acute gastroenteritis was 16.0% in total 268 tourists. Notably, the NoV RNA positive rate was 95.6% (22/23) in submitted specimens.

**Table 2:** Summary of investigation on NoV acute gastroenteritis outbreaks in tourist groups.

Event	Tourist origins	Expose date	Onset date	Numbers per group	Patient number	Prevalence rate (%)	Specimens numbers	Positive specimens	Genotypes	
A	Taiwan	Feb 8	Feb 9	25	5	20	2	2	GII	
B	Taiwan	Feb 8	Feb 9	33	6	18.2	2	2	GII	
C	Taiwan	Feb 9	Feb 10	16	4	25.0	1	1	GII	
D	Malaysia	Apr 10	Apr 11	22	5	22.7	4	4	GI.3	
E	Taiwan	Apr 12	Apr 13	31	3	9.7	4	3	GII.4, GII.6	
F	Taiwan	Apr15	Apr 16	38	6	15.8	4	4	GI.4, GII.4	
G	Taiwan	Apr 16	Apr 17	103	14	13.6	6	6	GI.3, GI.4, GII.14	
Total		/	/	268	43	16.0	22	23	GI.3, GI.4, GII.4, GII.6, GII.14,	
H*	Zhejiang	unknown	Apr 18	3	3	100	3	3	GI.3, GI.5, GII.6, GII.12	
	Employees**	unknown	/	/	/	/	14	1	GI.3	

* The exposure date for patients in Event H was between April 15 to April 17, 2012.

** indicate anal swabs from employees of the restaurant under investigation.

Despite diverse travel routes in the seven tourist groups’ agenda, one resort in common was frequently mentioned during the preliminary investigation. One day prior to their arrival at Xiamen, all of these groups visited a coastal resort, some 180 km away from Xiamen, had lunches in the same restaurant, where all of them ordered oyster pancakes prepared by fresh oyster with sweet potato starch. Therefore, an epidemiological investigation was launched for environment and employees of the restaurant on that island on April 18th. Field investigation showed the water supply system of the restaurant was constantly under risks of sewage contamination, while the restaurant continued its business as usual even in the wake of outbreaks in February. During laboratory tests, NoV RNA was also detected in one of several anal swabs collected from employees of that restaurant on April 18th. Hence, the serial outbreaks were linked to that restaurant. Three potential sources of NoV contamination were proposed, i.e., contaminated water supply, contaminated or undercooked foodstuff or healthy employees (chefs or waiters) as NoV carriers.

Meanwhile, another foodborne outbreak, in which three visitors from the neighboring Zhejiang province developed identical gastroenteritis symptoms in Xiamen, was reported on April 18th (Event H). During three days prior to onset, the visitors had fishes, bivalves, etc at several restaurants in Xiamen. Similarly, NoV RNA of GI and GII were identified in their feces. It was revealed by epidemiological investigation that NoV infections in Event H occurred solely in Xiamen.

### Negative Results for Other Pathogens or Specimens

No bacterial pathogen, including *Salmonella spp, Vibrio parahaemolyticus, Shigella spp, Staphylococcus aureus, Proteus spp* or diarrheagenic *Escherichia coli*, etc. was isolated from anal swabs or fecal specimens by conventional culture procedures. NoV RNA was not detected in any foodstuff or raw water samples.

### NoV RNA Detection in Patient Specimens

Among the 23 feces or anal swabs collected from patients of the serial outbreaks, one was negative in the real-time RT-PCR assay for NoV RNA, while the rest 22 specimens were positive, including 10 specimens positive for GI, 6 for GII, 6 for GI and GII simultaneously (Table 2). Particularly, positive specimens from Event A–C belonged to GII, specimens from Event D were positive for GI, however, co-infection of both GI and GII strains were identified in other specimens collected in April (Event E–G), as summarized in Table 2. These results indicated that diverse genogroups of NoV contributed to epidemic gastroenteritis outbreaks in the 7 tourist groups, and that patients from Event E–G were co-infected with GI and GII NoV. Notably, one anal swab from an employee of the restaurant under investigation, which was collected on the same day when patients in Event G were reported, was positive for GI. Meanwhile, both GI and GII strains were also circulating in Xiamen, as indicated by laboratory tests for Event H (Table 2).

### Sequence Analyses of Amplicons

In order to investigate genetic relationship between outbreak-associated NoV strains identified during the 70 days, DNA sequence data were attempted for each positive specimen. However, five specimens in early outbreaks (Event A–C) and another four specimens in April failed in RT-PCR because of low virus load (with Ct values greater than 33 in real-time PCR). In Event D–G, there were 13 amplicons obtained by RT-PCR amplification with virus-specific primers in 12 patients, including 9 GI sequences ranged from 271 to 285 bp and 4 GII fragments of 306–331 bp in length (Table 3). Meanwhile, one GI sequence was recovered from an employee of that restaurant, another five sequences were also obtained from three patients in Event H.

**Table 3:** List of positive specimens in NoV RNA detection and their genotypes.

Event	Specimen ID	Real-time PCR		Strain	Genotype	Accession number
		GI	GII			
A	S1	**−**	+	NT	NA	/
A	S2	**−**	+	NT	NA	/
B	S3	**−**	+	NT	NA	/
B	S4	**−**	+	NT	NA	/
C	S5	**−**	+	NT	NA	/
D	S6	+	**−**	NT	NA	/
D	S7	+	**−**	NT	NA	/
D	S8	+	**−**	NT	NA	/
D	S9	+	**−**	NVxm001	GI.3	KC783710
E	S10	+	+	NT	NA	/
E	S11	**−**	+	NVxm015	GII.4	KC783224
E	S12	+	+	NVxm016	GII.6	KC783725
F	S13	+	**−**	NT	NA	/
F	S14	+	**−**	NVxm002	GI.4	KC783711
F	S15	+	**−**	NVxm003	GI.4	KC783712
F	S16	+	+	NVxm014	GII.14	KC783723
				NVxm004	GI.4	KC783713
G	S17	+	+	NVxm017	GII.14	KC783726
G	S18	+	**−**	NVxm005	GI.3	KC783714
G	S19	+	+	NVxm006	GI.3	KC783715
G	S20	+	**−**	NVxm007	GI.4	KC783716
G	S21	+	+	NVxm008	GI.3	KC783717
G	S22	+	**−**	NVxm009	GI.4	KC783718
H	S23	+	+	NVxm010	GI.3	KC783719
				NVxm018	GII.6	KC783727
H	S24	+	+	NVxm011	GI.3	KC783720
				NVxm019	GII.12	KC783728
H	S25	+	**−**	NVxm012	GI.5	KC783721
employee	S26	+	**−**	NVxm013	GI.3	KC783722

NT, not tested; NA, not available.

It was revealed by BLAST analyses on NCBI website that these 19 NoV fragments (accession numbers: KC783710–KC783928) were classified into seven genotypes including GI.3, GI.4, GI.5, GII.4, GII.6, GII.12 and GII.14, and that serial outbreak associated genotypes were GI.3, GI.4, GII.4, GII.6 and GII.14. As shown in Table 3, one GI.3 sequence was obtained from 4 specimens in Event D. One GII.4 and one GII.5 fragments were identified in 3 specimens from Event E, in which another 2 samples were GI RNA positive in real-time PCR assays but failed in amplification of VP1 fragments. Two GI.4 sequences and one GII.14 sequence were identified in 4 specimens collected in Event F. As for Event G, five NoV sequences were obtained from 6 specimens, including 2 GI.3 strains, 2 GI.4 strains and one GII.14 strain. On the other hand, the NoV strain from a restaurant employee belonged to GI.3, while one GII.6, one GII.12 and one GI.5 strain, respectively, and another two GI.3 strains were detected from 3 specimens in Event H (Table 3).

Preliminary sequence alignment showed that 6 GI.3 strains were identical in the nt5387–5657 region within the genotype, and that another 5 GI.4 strains were identical in the same region, however, a total of 59 bp dispersed variation were observed between GI.3 and GI.4 strains. In general, lower homology was confirmed between other GI and GII sequences. Meanwhile, since it was suggested by epidemiological investigation that patients in Event H did not visit the coastal resort and the restaurant as other groups did, nucleotide similarity between sequences in Event H and those in other outbreaks was particularly concerned. Sequence alignment indicated a total of 67 bp variation in the nt5398–5635 region of Event H-associated strains (NVxm010(GI.3), NVxm011(GI.3) and NVxm012(GI.5)), slightly lower than sequence similarities of other outbreaks, although greater than 85% in homology were observed in comparison with sequences of relevant genotypes in GenBank. Therefore, in order to address the genetic relationship between these strains and circulating NoV strains worldwide, ten reference sequences with high nucleotide similarity and distinct geographic origins, were downloaded to generate a phylogenetic tree. Meanwhile, since 4 genotypes of NoV were detected in specimens from the three patients, phylogenetic analysis of sequence data from previous outbreaks with those in Event H, as representatives of local circulating strains, would differentiate NoV of diverse origins, especially from those in Event A–G.

**Figure 1:**
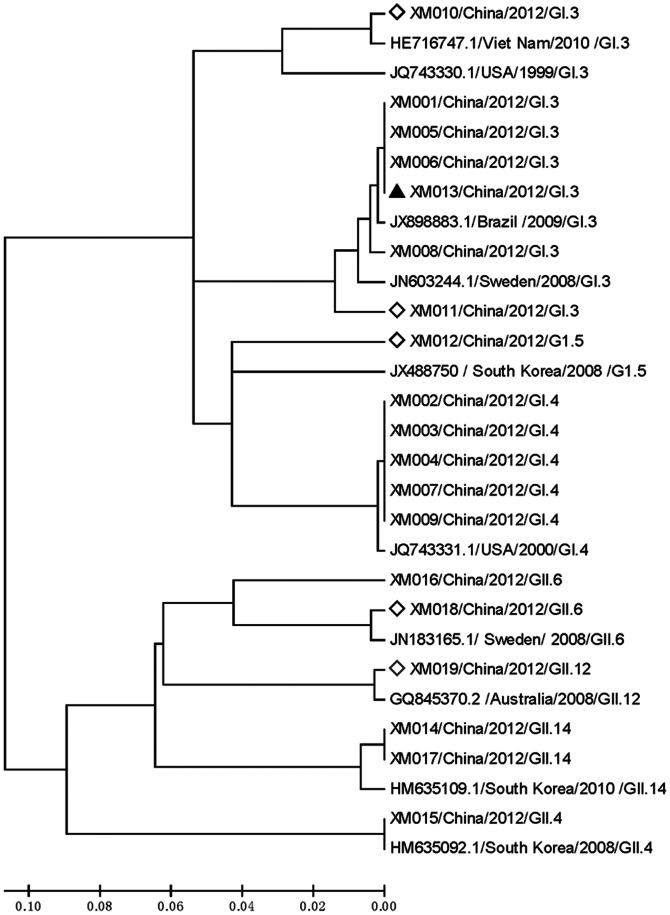
Phylogenetic analyses of partial RdRP/VP1 genes in NoV strains associated with serial foodborne outbreaks in tourist groups. Thirteen strains belonging to five genotypes including GI.3, GI.4, GII.4, GII.6 and GII.14 were detected in 22 specimens from Event A–G., which was associated with infections occurred at the restaurant. One GI.3 strain (labeled with black triangle) was detected in one restaurant employee. Local infections with 4 genotypes (labeled with diamond) such as GI.3, GI.5, GII.6 and GII.12 were identified in 3 patients.

It was implied from Figure 1, based on one GI.3 strain cluster (NVxm006, NVxm013, NVxm005, NVxm001 and NVxm008), one GI.4 strain cluster (NVxm002, NVxm003, NVxm004, NVxm007 and NVxm009) and another GII.14 cluster (NVxm014, NVxm017), that there was close genetic relationship between the outbreak-associated NoV strains, suggesting these serial NoV outbreaks were linked to that restaurant. On the contrary, NoV sequences obtained in Event H were distinct from sequences within these clusters, either in outbreak-associated genotypes such as GI.3 (NVxm010 and NVxm011) and GII.6 (NVxm018), or unique genotypes including GI.5 (NVxm012) and GII.12 (NVxm019) solely observed in Event H. As several genotypes were identified by nucleotide analysis of sequences obtained in the outbreaks, it was concluded from epidemiological investigation and laboratory analyses that multiple genotypes of NoV contributed to the serial foodborne outbreaks in tourists in Xiamen, 2012.

## Discussion

Combined with description of clinical symptoms, epidemiological investigation and laboratory tests, the 7 acute gastroenteritis outbreaks in tourist groups in Xiamen could be classifies as multiple genotypes of NoV caused foodborne gastroenteritis, since co-infection with multiple NoV genotypes occurred at least in three groups of 172 tourists. Based on the following evidence chain, it could be concluded that Event A–G were attributed to acute NoV infection when each group had lunch in that restaurant: 1) Most specimens collected in Event A–G were positive for NoV RNA, but negative for other common foodborne bacterial pathogens; 2) Tourists in Event A–G had lunches in the same restaurant one day prior to onset, visited hospitals in Xiamen on the next day. The estimated incubation period was consistent with that of NoV ranging from 12 to 48 hours [7]. 3) The GI.3 strain recovered from an anal swab of one restaurant employee was identical to some patient strains in Event D–G. 4) Five NoV sequences from local-infected patients in Event H were distinct from those in Event A–G.

The globally wide-spread NoV strains are characterized by their genetic diversities with dozens of genotypes within currently known five genogroups and frequently emerging recombination variants [17]. As a worldwide dominant genogroup, GII strains are contributed to the majority of viral gastroenteritis, in particular, GII.4 strains have caused at least 4 pandemic seasons since late 1990s, also as one of the predominant genotypes detected in China [17]–[21]. There were seven genotypes identified in these serial outbreaks, although only one GII.4 strain was detected in one specimen from Event E, yet the role of GII.4 stains could not be neglected, since amplification of conventional RT-PCR for GII fragments was unsuccessful in several co-infected cases, and causative agents in Event A–C belonged to GII as well. At least 8 patients were co-infected with GI and GII strains during the investigation, indicating co-infection are responsible for a considerable proportion of NoV infections, although it is not clear whether simultaneously co-infection of two genogroups may worsen the outcome of infection or facilitate virus recombination. Meanwhile, unlike patients in Event A–G with a common source of infection, the three patients in Event H had meals in several restaurants in Xiamen, their NoV sequences and genotypes differed from those in Event A–G, suggesting multiple NoV genotypes were also co-circulating in Xiamen at that time.

Previous observation revealed that NoV illness can resolve within 48 hours, while virus shedding can be prolonged for several weeks [3]. There was a 70-day span during the serial outbreaks, at least 43 tourists out of 268 developed enteric illness after having lunches in that restaurant, suggesting there were certain persistent sources of contamination in the restaurant such as contaminated water supply, contaminated foodstuff, or employees as NoV carriers. It was indicated from epidemiological investigation that this restaurant mainly took water from a self-owned well in its food processing, and that only one employee was sick from diarrhea during February and April. Since multiple genotypes were detected in Event D–G, the employee, whose specimen was identified containing a GI.3 strain, was not the sole source of contamination. Comparison with menus at that restaurant in Event A–G suggested that oyster pancakes were served in every group, thus, raw or undercooked oyster was probably another source of NoV contamination, although food specimens collected on April 18 at the restaurant were negative for NoV RNA. Meanwhile, field investigation showed the main source of water supply in the restaurant was potentially contaminated in several locations (unpublished data). Therefore, their water supply system was likely another persistent contamination source of NoV.

NoV-associated acute gastroenteritis outbreaks are common in closed or semi-closed settings such as cruise ships, military camps, schools or elder care homes, etc, however, most outbreaks are controllable when proper intervention measures are taken immediately, such serial outbreaks lasting over 2 months are rarely reported [22], [23]. During laboratory analyses on early outbreaks in February, fecal specimens were only tested for NoV RNA, sequence analyses were not attempted in routine surveillance, thus relevant sequences were absent in current phylogenetical analysis. In principle, real-time PCR assays cannot distinguish infection origins of patients in Event A–G from that in Event H, nevertheless, sequence analyses of amplicons available finally linked the outbreaks to a restaurant some 180 km away. Since molecular surveillance of non-cultivable NoV at the nucleotide level can provide with direct evidence in tracking the source of outbreaks, such strategy will be encouraged to better identify risks in future investigations.

In conclusion, the aforementioned serial foodborne NoV outbreaks in tourist groups in Xiamen, 2012, were caused by multiple genotypes linked to a restaurant. Extensive epidemiological investigation and sequence analyses played important roles in exploring probable clues for the serial NoV outbreaks of unknown origins and directing appropriate timely intervention strategies.
